# A Rare Presentation of Severe Hypothyroidism With Catatonia and Psychosis: Myxedema Madness

**DOI:** 10.7759/cureus.106161

**Published:** 2026-03-30

**Authors:** Ayedh H Al Ghamdi, Reem A Alharbi, Amjad J Alfaifi, Najd A Bin Tuwalah

**Affiliations:** 1 Department of Psychiatry, College of Medicine, King Saud University, Riyadh, SAU; 2 Department of Psychiatry, King Khalid University Hospital, Riyadh, SAU; 3 Department of Psychiatry, Ministry of Health, Riyadh, SAU; 4 Department of Medicine, Dar Al Uloom University, Riyadh, SAU

**Keywords:** catatonia, hypothyroidism, myxedema, neuropsychiatric manifestations, psychotic disorders, thyroid hormone replacement

## Abstract

Myxedema psychosis or “myxedema madness” is a rare, treatable, but potentially fatal neuropsychiatric manifestation of severe hypothyroidism. This case report presents a 46-year-old, unemployed, childless woman with progressive deterioration of her health over the last six months, including progressive lethargy, social withdrawal, poor intake, irritability, sound sensitivity, auditory hallucinations, urine and fecal incontinence, and complete neglect of personal hygiene. She had undergone total thyroidectomy and parathyroidectomy for which she was on levothyroxine, which she had discontinued. On physical examination, she was disoriented, having disorganized thinking, false belief of having cancer, fecal soiling, non-pitting edema of the lower limbs, and rigidity of the upper and lower limbs. Laboratory investigations showed hypoglycemia, hypokalemia, hypophosphatemia, severe hypothyroidism, and low morning cortisol. The CT scan of the brain revealed symmetrical calcification of bilateral basal ganglia, thalami, right corona radiata, and bifrontal subcortical white matter. She was started on levothyroxine, hydrocortisone, and aripiprazole. On day 16, she developed catatonic features, for which she was treated with lorazepam and aripiprazole, resulting in gradual improvement.

## Introduction

Hypothyroidism is a common endocrine disorder characterized by insufficient thyroid hormone production and symptoms, such as fatigue, cold intolerance, weight gain, constipation, and dry skin, while in severe cases, it may present with myxedema psychosis or myxedema coma [1,2]. Psychosis with catatonia or "myxedema madness" is a rare, treatable but potentially fatal neuropsychiatric manifestation of hypothyroidism [3]. Neuropsychiatric features of hypothyroidism include apathy, depression, psychosis, cognitive decline, dementia, catatonia, and affective disorders [4,5]. Psychosis is an amalgamation of symptoms, such as general confusion, disorientation, bouts of restless violence, delusions, hallucinations, and disorganized thinking, resulting in loss of contact with reality [6]. Catatonia refers to a neuropsychiatric syndrome encompassing hyperactivity manifestations to hypoactivity states, including mutism, stupor, catalepsy, waxy flexibility, negativism, stereotypes, mannerisms, grimacing, agitation, and echophenomena [7-9]. The co-existence of hypothyroidism and psychiatric disorders is attributed to the genetic correlations and shared biological pathways [10]. Although psychiatric features are frequently experienced by hypothyroid patients, only a few case reports are available in the literature on hypothyroidism with catatonia and psychosis. Therefore, this case report of a female patient having severe hypothyroidism manifesting as catatonia and psychosis is a useful addition to the literature.

## Case presentation

A 46-year-old, unemployed, childless woman with a primary school education was brought to the emergency department by her husband due to progressive deterioration of her health over the preceding six months. Her husband reported that she had undergone total thyroidectomy and parathyroidectomy years ago at another hospital and had been maintained on levothyroxine. Recently, she discontinued her thyroid replacement therapy for an unknown period. According to the patient’s husband, the patient had progressive functional decline over several months with poor self-care and limited social support, which likely contributed to neglect of her prescribed levothyroxine therapy. This was followed by progressive lethargy, social withdrawal, poor intake, irritability, sound sensitivity, and auditory hallucinations with behavioral responses to unseen stimuli. She developed urinary and fecal incontinence and complete neglect of her personal hygiene. On examination, her vital signs were stable. She was confused with a disorganized thought process, with clothing soiled with feces, non-pitting edema involving all upper and lower extremities, more pronounced in the lower limbs, and rigidity of both upper and lower limbs. Respiratory, cardiovascular, and gastrointestinal examinations were unremarkable.

Laboratory investigations revealed hypoglycemia (blood glucose 2.69 mmol/L), hypokalemia (K = 3.07 mmol/L), and hypophosphatemia (0.69 mmol/L). Cerebrospinal fluid analysis was within normal limits (Table 1).

**Table 1:** Laboratory investigations TSH: thyroid-stimulating hormone; T4: thyroxine

Test	Result	Unit	Reference Range
Blood glucose	2.69	mmol/L	3.9-5.5
Potassium (K)	3.07	mmol/L	3.5-5.1
Phosphate	0.69	mmol/L	0.8-1.5
TSH	199	mIU/L	0.4-4.0
Free T4	4.8	pmol/L	12-22
Morning cortisol	66.8	nmol/L	140-690
CSF analysis	Normal	-	Normal

**Figure 1:**
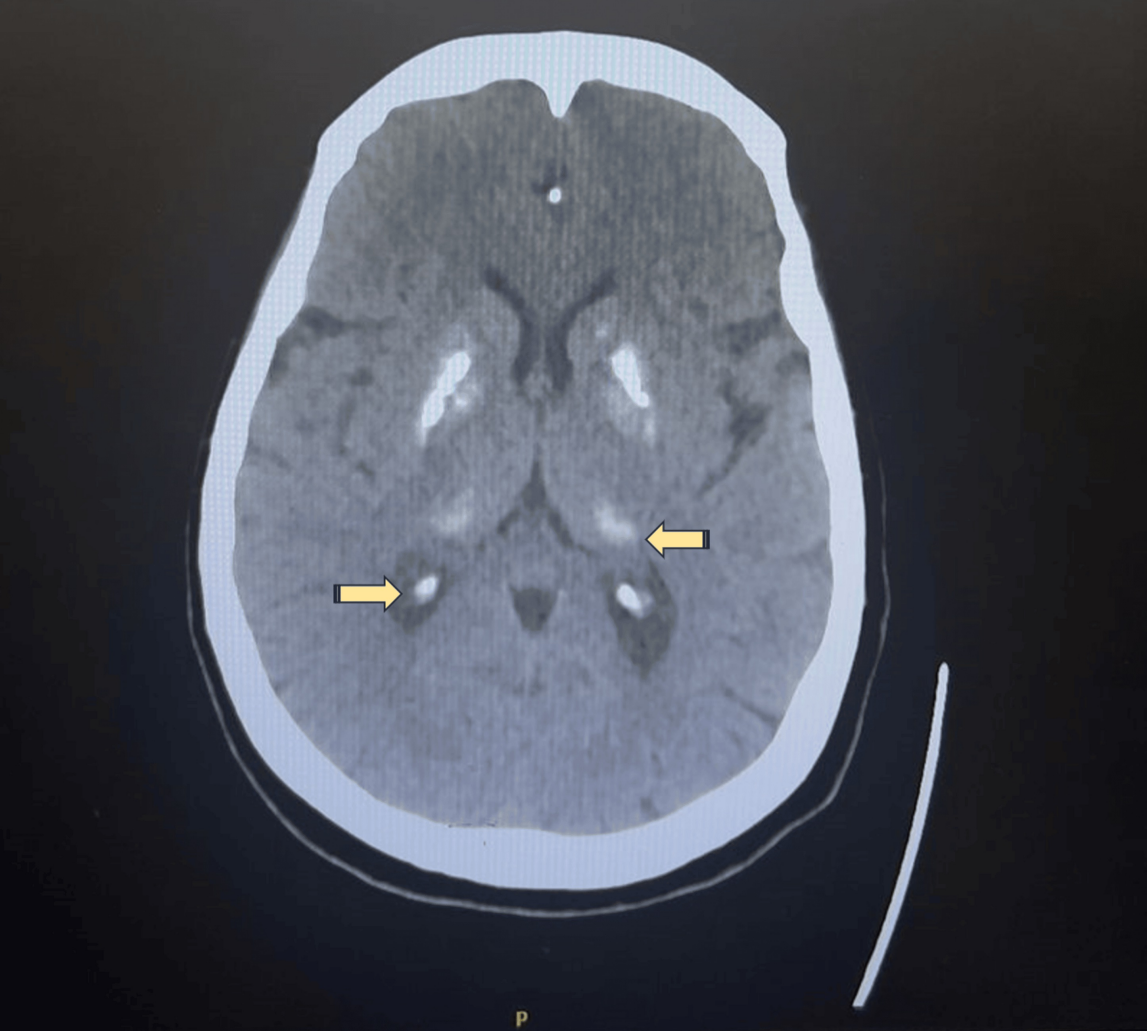
CT of the brain

Brain CT scan revealed symmetrical calcification of the bilateral basal ganglia, thalami, right corona radiata, and bifrontal subcortical white matter, as shown in Figure 1.

**Figure 2:**
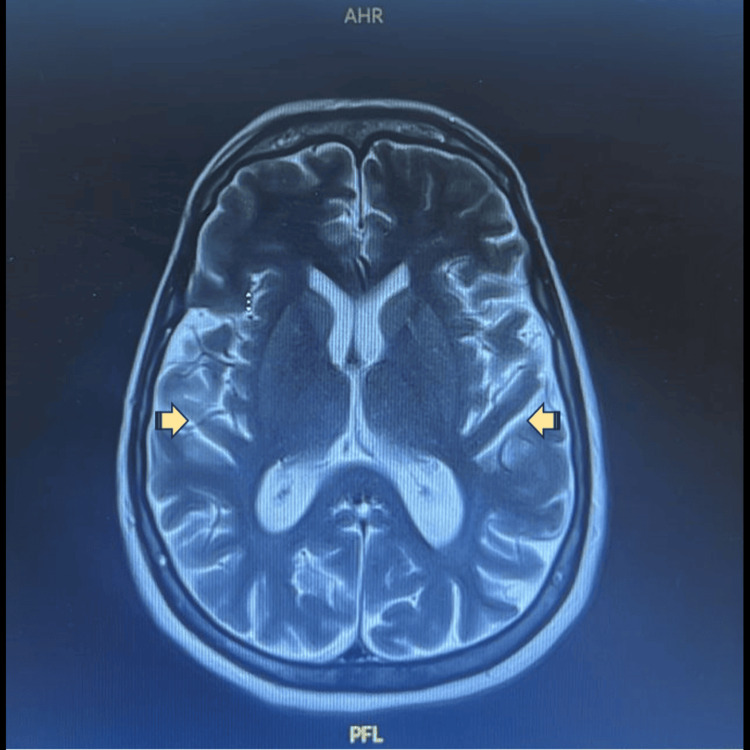
MRI of the brain

MRI of the brain showed bilateral basal ganglia and subcortical calcifications, with diffuse mild to moderate brain atrophic changes, raising the possibility of Fahr syndrome, as shown in Figure 2.

She was admitted under neurology with a differential diagnosis of Fahr disease, metabolic disorders, Wilson disease, or primary psychiatric illness. Endocrinology consultation was obtained following abnormal thyroid and adrenal function tests, which demonstrated markedly elevated thyroid-stimulating hormone (TSH) (199 mIU/L), low T4 (4.8 pmol/L), and low morning cortisol (66.8 nmol/L). Based on clinical examination and investigations, levothyroxine and hydrocortisone replacement therapy were initiated.

On the second day of admission, a psychiatric evaluation was conducted. The patient was uncooperative and agitated. She was disoriented to time, place, and person, stared blankly, responded inappropriately to questions, interacted with unseen stimuli, and expressed fear of having cancer. She was started on intramuscular haloperidol 5 mg at bedtime, which was subsequently reduced to 2.5 mg. However, treatment was discontinued after two days due to QTc prolongation on ECG (568 ms). During the second week of hospitalization, the patient developed prominent catatonic features, including immobility, persistent forward neck flexion, mutism, poor eye contact, severe plantarflexion contractures, and cogwheel rigidity of the upper limbs. At that time, thyroid function tests showed persistent biochemical hypothyroidism despite initial treatment (TSH 145 mIU/L, free T4 9.32 pmol/L). The Bush-Francis Catatonia Rating Scale (BFCRS) [11] score was 5. A lorazepam challenge test (1 mg orally) was performed, which resulted in partial improvement in speech, interaction, and upper limb movement. The dose was gradually titrated to 1 mg in the morning and 2 mg at night, leading to complete resolution of catatonic symptoms. Aripiprazole was initiated at 5 mg and gradually titrated to 20 mg over four weeks for persistent psychotic symptoms. During this period, the patient demonstrated gradual but substantial improvement. At the time of reporting, she showed good recovery, with complete resolution of psychotic and catatonic features and no adverse effects from treatment reported. 

## Discussion

Although patients suffering from hypothyroidism frequently experience neuropsychiatric manifestations. However, psychosis with catatonia or "myxedema madness" is a rare complication of severe untreated hypothyroidism [12]. Psychosis was described as a complication of hypothyroidism in 1883 by the Committee of the Clinical Society of London [13]. Reported that catatonia occurs in 7-38% of psychiatric patients [14]. They found that psychosis occurs in 5-15% of hypothyroid patients [15]. The current presentation of both psychosis and catatonia in hypothyroidism is extremely rare, with fewer cases reported in the literature.

The term "myxedema madness" was first coined by Asher (1949) when he reported 14 patients with myxedema and psychosis [16]. Since then, several cases of myxedema madness have been reported in the literature [12,13,17].

In this report, despite having a record of total thyroidectomy and parathyroidectomy, the patient quit levothyroxine for an unknown duration, resulting in a crisis of severe hypothyroidism and a challenging diagnosis of myxedema madness. Documents rarely reported extreme levels of TSH (199 mIU/L) and T4 (4.8 pmol/L) in a living patient, contributing to the understanding of severe endocrine pathology and its profound impact on mental health. Interestingly, psychosis may be manifested in patients with both overt (clinical) or subclinical hypothyroidism, highlighting the importance of considering hypothyroidism in the cases of new-onset psychosis [18,19]. Although myxedema madness occurred in severe hypothyroidism in the present case, it may be the initial symptom of hypothyroidism [20]. Gondwal et al. reported psychosis as an acute presentation of Hashimoto's thyroiditis. Hence, it is important to know that psychosis may occur in primary hypothyroidism (subclinical or clinical), Hashimoto's thyroiditis, and hypothyroidism after thyroidectomy [21]. Conversely, neuropsychiatric features such as catatonia have also been reported in patients suffering from hyperthyroidism [22-24], which should not be mixed with myxedema madness. Hence, catatonia can be manifested by both hypothyroid and hyperthyroid patients, which further indicates a complex and integrated association between thyroid diseases and psychiatric disorders. In this context, acute psychosis may result in a missed diagnosis of hypothyroidism, which may lead to serious outcomes [25].

Treatment of psychosis with catatonia in hypothyroidism or myxedema madness includes lifelong thyroid replacement therapy and antipsychotic drugs at the start. There are currently no established guidelines defining the optimal duration of antipsychotic therapy in myxedema psychosis. Based on the available literature, antipsychotics are generally used as short-term adjunctive therapy to control severe psychotic symptoms until thyroid hormone replacement leads to clinical improvement, after which they are gradually tapered or discontinued [13,19,26]. Barata et al. [19] treated a 46-year-old woman suffering from myxedema psychosis with oral thyroxine (150 mcg and titrated to 250 mcg per day at day 3) and olanzapine (10 mg per day), which resulted in rapid improvement and complete remission of symptoms in 10 days. They discontinued olanzapine after three months with sustained remission of psychotic symptoms for two years of follow-up [19]. Sardar et al. [25] treated a 36-year-old lady suffering from myxedema madness with oral thyroxine (100 mcg/day), fluoxetine (20 mg daily), and haloperidol as needed for the short term, which settled symptoms within days [25]. Omri et al. [27] presented a 60-year-old woman with new-onset psychosis after cessation of levothyroxine post-subtotal thyroidectomy. She showed no response to initial doses of lorazepam and olanzapine but improved with thyroxine therapy on the diagnosis of hypothyroidism. Thus, the treatment of myxedema psychosis mainly includes oral thyroxine therapy and short-term use of antipsychotics [27]. In this case study, we selected aripiprazole as the antipsychotic for our patient because of many important safety concerns. First, hypothyroidism may prolong the QTc more likely by altering the function of cardiac ion channels and inducing metabolic disturbances [2,15]. Our patient had severe QTc prolongation (568 ms) on haloperidol, necessitating immediate discontinuation. Compared with most atypical and typical antipsychotics, aripiprazole has a much lower risk of QTc prolongation [23,24]. Second, aripiprazole has a favorable metabolic profile [4]. This feature is important for patients with endocrine dysfunction. Third, aripiprazole, as a partial dopamine D2 agonist, will effectively manage psychotic symptoms in addition to reducing extrapyramidal side effects [8].

To the best of our knowledge, based on the currently available literature, this may represent the first reported use of aripiprazole in hypothyroidism-induced catatonia and psychosis. Previous case reports have recorded the administration of haloperidol [7], olanzapine [8], and quetiapine [5] in similar presentations; however, none have documented the use of aripiprazole in this particular context. Our patient's positive outcome, characterized by the full remission of psychotic and catatonic symptoms after four months, indicates that aripiprazole may represent a potential option for similar conditions. Further research should be considered in this regard.

This case is unique for several reasons related to its rarity and novelty. First, psychosis with catatonia in hypothyroidism (myxedema madness) is exceedingly rare in the medical literature, with only a few cases reported to date and no published case series [13,26]. Second, to the best of our knowledge, this is the first reported case in the Middle East region and Saudi Arabia. Third, based on the currently available literature, this may represent one of the earliest reported uses of aripiprazole for hypothyroidism-induced catatonia and psychosis, contributing to the limited body of literature on the management of similar cases.

Furthermore, this case illustrates a diagnostic dilemma involving overlap among endocrinological, psychiatric, and neurological conditions. The patient's presentation initially proposed primary psychiatric illness, and without complete endocrine evaluation, could easily be misdiagnosed as first-episode psychosis or catatonic disorder, emphasizing the importance of evaluating all these systems in cases of first-episode psychosis. This supports the argument that psychiatrists should routinely investigate thyroid function in patients presenting with first-episode schizophrenia. Our case highlights several critical red flags that maintain a high clinical suspicion for organic etiologies in psychiatric presentations, particularly late age of first psychiatric symptoms (age 46 in our patient), rapid symptom progression over weeks to months, prominent cognitive impairment and disorientation, autonomic instability, bowel and bladder incontinence, and resistance to standard psychiatric interventions. The presence of many red flags should encourage a thorough medical investigation, despite the patient's psychiatric symptoms. Given the lack of guidelines in the literature for dealing with life-threatening complications like this case, there is an emphasis on the need for greater awareness and education about these rare but reversible conditions.

Beyond the psychiatric presentation, neuroimaging findings in this case provided additional diagnostic complexity. The CT scan revealed symmetrical calcification of bilateral basal ganglia, thalami, right corona radiata, and bifrontal subcortical white matter, which was consistent with a diagnosis of Fahr syndrome, manifesting with various neuropsychiatric and cognitive symptoms [28]. Fahr syndrome is a rare neurological disorder characterized by bilateral intracranial calcifications, most commonly involving the basal ganglia. These calcifications may also affect other brain regions and are typically identified on neuroimaging studies. The clinical presentation is heterogeneous and may include neurological and neuropsychiatric manifestations. Reported psychiatric manifestations include psychosis, cognitive impairment, mood disturbances, and behavioral changes [28]. Hence, these radiological findings posed a diagnostic challenge, as similar intracranial calcifications have been described in Fahr syndrome, which has been reported in association with hypothyroidism [29,30].

This case has significant educational value and lays the foundation for future studies. Furthermore, the optimal choice, dosing, and duration of antipsychotic therapy in myxedema psychosis requires further exploration. Our experience with aripiprazole suggests that future studies comparing antipsychotics for safety and effectiveness in hypothyroid patients may be warranted. Finally, anticipating and recognizing patients at the highest risk for developing myxedema psychosis could help in starting prevention measures and monitoring, potentially avoiding the occurrence of life-threatening complications. By increasing the understanding of this rare but reversible condition through case documentation, we hope to facilitate earlier detection, management, and improved outcomes for patients worldwide.

## Conclusions

This case highlights the need for a thorough medical evaluation in patients demonstrating a first-episode psychosis and catatonia. should consider hypothyroidism as a reversible cause of neuropsychiatric presentations. Earlier detection and suitable endocrine and psychiatric management are essential to prevent dangerous complications. In addition, this report suggests that aripiprazole may represent a safe and practical therapeutic option in such cases. Additional studies are required to establish optimal management approaches.

## References

[1] Kaplan JL, Castro-Revoredo I (2020). Severe hypothyroidism manifested as acute mania with psychotic features: a case report and review of the literature. J Psychiatr Pract.

[2] Zamwar UM, Muneshwar KN (2023). Epidemiology, types, causes, clinical presentation, diagnosis, and treatment of hypothyroidism. Cureus.

[3] Konda PR, Reddy S, Gunde S (20231). A rare case of recurrent psychosis with hypothyroidism precipitating catatonia. Telangana J Psychiatry.

[4] Lekurwale V, Acharya S, Shukla S, Kumar S (2023). Neuropsychiatric manifestations of thyroid diseases. Cureus.

[5] Woody DM, Chen C, Parker J (2023). Catatonia in a patient with bipolar affective disorder and hypothyroidism: a diagnostic and therapeutic challenge. Cureus.

[6] Calabrese J, Al Khalili Y (2026). Psychosis. StatPearls.

[7] Iskandar M, Stepanova E, Francis A (2014). Two cases of catatonia with thyroid dysfunction. Psychosomatics.

[8] Edinoff AN, Kaufman SE, Hollier JW (2021). Catatonia: clinical overview of the diagnosis, treatment, and clinical challenges. Neurol Int.

[9] Phiri P, Delanerolle G, Hope O, Murugaiyan T, Dimba G, Rathod S, Zingela Z (2024). Catatonia: a deep dive into its unfathomable depths. World J Psychiatry.

[10] Zhou J, Zhu L (2024). Shared genetic links between hypothyroidism and psychiatric disorders: evidence from a comprehensive genetic analysis. Front Endocrinol (Lausanne).

[11] Bush G, Fink M, Petrides G, Dowling F, Francis A (1996). Catatonia. I. Rating scale and standardized examination. Acta Psychiatr Scand.

[12] Bhattarai HB, Kunwar GJ, Rijal A (2022). Acute psychosis unveiling diagnosis of hypothyroidism: a case report. Ann Med Surg (Lond).

[13] Krüger J, Kraschewski A, Jockers-Scherübl MC (2021). Myxedema madness - systematic literature review of published case reports. Gen Hosp Psychiatry.

[14] Ahmed A, Patil R, Longkumer I (2023). A case of acute catatonia precipitated by psychosis successfully treated with lorazepam: a case report. Cureus.

[15] Rasmussen MJ, Robbins H, Fipps DC, Rustad JK, Pallais JC, Stern TA (2023). Neuropsychiatric sequelae of thyroid dysfunction: evaluation and management. Prim Care Companion CNS Disord.

[16] Asher R (1949). Myxoedematous madness. Br Med J.

[17] Yahaya NS, Borhan MK (2025). Myxoedema madness: when hypothyroidism turns psychotic. J ASEAN Fed Endocr Soc.

[18] Şahin F, Arıkan Z (2021). Ego-dystonic psychotic symptoms as the initial manifestations of hypothyroidism in an elderly man. Turkiye Klin J Case Rep.

[19] Barata VA, Bastos J, Felício R, Cativo C, Gonçalves P (2023). Case report: myxedema psychosis caused by subclinical hypothyroidism. Psychiatry Res Case Rep.

[20] Ueno S, Tsuboi S, Fujimaki M, Eguchi H, Machida Y, Hattori N, Miwa H (2015). Acute psychosis as an initial manifestation of hypothyroidism: a case report. J Med Case Rep.

[21] Gondwal R, Avinash PR, Pal A, Modi S (2021). Psychosis as acute presentation in Hashimoto's thyroiditis. Indian J Psychiatry.

[22] Sharma P, Charkhe P, Mahesh Kumar (2022). Catatonia associated with thyrotoxicosis. Indian J Psychiatry.

[23] Ressler HW, Shah K, Quattlebaum T, Munjal S (2023). Catatonia and psychosis associated with hyperthyroidism. Prim Care Companion CNS Disord.

[24] Chaikind JR, Pambianchi HL, Bledowski C (2025). Catatonia associated with hyperthyroidism: an illustrative case and systematic review of published cases. J Acad Consult Liaison Psychiatry.

[25] Sardar S, Habib MB, Sukik A (2020). Myxedema psychosis: neuropsychiatric manifestations and rhabdomyolysis unmasking hypothyroidism. Case Rep Psychiatry.

[26] Mohamed MF, Danjuma M, Mohammed M (2021). Myxedema psychosis: systematic review and pooled analysis. Neuropsychiatr Dis Treat.

[27] Omri M, Ferhi M, Lentz N, Oliveira Galvao M, Hamm O (2024). Myxedema psychosis: diagnostic challenges and management strategies in hypothyroidism-induced psychosis. Cureus.

[28] Wazir MH, Ali Y, Mufti AZ, Ahmad A, Ahmad H (2023). Fahr’s syndrome: a rare case presentation. Cureus.

[29] Malik N, Pattan V, Nai Q, Yousif A (2014). Hypoparathyroidism, hypothyroidism and thrombocytopenia: rare constellation of Fahr’s syndrome. J Endocrinol Metab.

[30] Kumar S, Gaad AA, Irshad Abbasi M, Afzal M, Shah S, Kumar D (2018). Association of Fahr disease with rhabdomyolysis and hypoparathyroidism. Pak J Neurol Sci.

